# Scanning fast and slow: current limitations of 3 Tesla functional MRI and future potential

**DOI:** 10.3389/fphy.2014.00001

**Published:** 2014-02-11

**Authors:** Roland N. Boubela, Klaudius Kalcher, Christian Nasel, Ewald Moser

**Affiliations:** 1 Center for Medical Physics and Biomedical Engineering, Medical University of Vienna, Vienna, Austria; 2 MR Center of Excellence, Medical University of Vienna, Vienna, Austria; 3 Department of Radiology, State Clinical Center Danube District, Tulln, Austria; 4 Brain Behavior Laboratory, Department Psychiatry, University of Pennsylvania Medical Center, Philadelphia, PA, USA

**Keywords:** fMRI, resting state, sensitivity, specificity, speed, physiological noise

## Abstract

Functional MRI at 3T has become a workhorse for the neurosciences, e.g., neurology, psychology, and psychiatry, enabling non-invasive investigation of brain function and connectivity. However, BOLD-based fMRI is a rather indirect measure of brain function, confounded by physiology related signals, e.g., head or brain motion, brain pulsation, blood flow, intermixed with susceptibility differences close or distant to the region of neuronal activity. Even though a plethora of preprocessing strategies have been published to address these confounds, their efficiency is still under discussion. In particular, physiological signal fluctuations closely related to brain supply may mask BOLD signal changes related to “true” neuronal activation. Here we explore recent technical and methodological advancements aimed at disentangling the various components, employing fast multiband vs. standard EPI, in combination with fast temporal ICA. Our preliminary results indicate that fast (*TR* <0.5 s) scanning may help to identify and eliminate physiologic components, increasing tSNR and functional contrast. In addition, biological variability can be studied and task performance better correlated to other measures. This should increase specificity and reliability in fMRI studies. Furthermore, physiological signal changes during scanning may then be recognized as a source of information rather than a nuisance. As we are currently still undersampling the complexity of the brain, even at a rather coarse macroscopic level, we should be very cautious in the interpretation of neuroscientific findings, in particular when comparing different groups (e.g., age, sex, medication, pathology, etc.). From a technical point of view our goal should be to sample brain activity at layer specific resolution with low TR, covering as much of the brain as possible without violating SAR limits. We hope to stimulate discussion toward a better understanding and a more quantitative use of fMRI.

## Background

Hampered by the inherently low sensitivity, caused by low energy difference in MRI spin transitions (ca.10^−6^eV), and the low speed of data collection in 2D/3D MRI, increasing sensitivity was the prime focus for decades. This led to the development of a versatile diagnostic imaging technique, based on morphological imaging, using endogenous (e.g., based on tissue relaxation times T_1_, T_1ρ_, T_2_, T_2_*) or exogenous tissue contrast during the 1980’s. Later on, employing fast gradient-echo imaging techniques, functional imaging based on blood-oxygenation [1] and perfusion [2] changes have been developed. However, substantially increasing image SNR via stronger static magnetic fields, and the sensitivity of multi-element phased-array coils and corresponding accelerated imaging acquisition techniques, did not yet increase time series SNR [3] nor, subsequently, contrast in functional MRI (fMRI). This is mainly due to non-white physiological noise, varying in different brain regions, and interacting with signal reduction due to susceptibility differences between brain tissue, cerebro-spinal fluid (CSF), air and bone as well as gross head motion. We are convinced, and will demonstrate below, that time has come to trade sensitivity for specificity in functional MRI of the human brain.

Owing to the rapid technical developments in magnet and rf-technology, increasing sensitivity via the increased field strength of the static magnetic field and the efficiency of rf-coils, as well as to its increasing value in clinical diagnosis and basic research, magnetic resonance imaging (MRI) and spectroscopy (MRS) show a very dynamic course for over 30 years. Furthermore, a plethora of rf-excitation and readout protocols as well as reconstruction algorithms help to employ the still limited tissue magnetization ever more efficiently and, thus, to speed up (spectroscopic) imaging techniques. In blood oxygenation level-dependent (BOLD) fMRI, developed over 20 years ago (for a review see 4), it was quite clear from the beginning that the method may be compromised by artifact signals from head motion [5, 6] and physiology (7–14, etc.), leading to a “brain or vein?” discussion [15–17]. In 1995, Biswal et al. [79] opened up a new field, termed resting-state fMRI, by correlating spontaneous (i.e., measured in the absence of any specific task) signal fluctuations in different brain areas. Starting from a freely chosen seed-region, “resting-state” networks were obtained by correlating the time courses of the seed and other regions, presumably characterized by very slow signal fluctuations (<0.1 Hz). After the introduction of exploratory fMRI analysis [18, 19] model free analysis of spontaneous and task related BOLD-signal fluctuations became available [20–23], avoiding rigid modeling of a complex biological system. On the other hand, a wide (frequency) range of noise sources might mask “true,” i.e., neuronal activity related, connectivity [14, 24–27]. Using short *TR* single slice echo-planar imaging (EPI), it has been shown that high-frequency fluctuations resulting from heart-beat driven physiology may coexist with very slow signal fluctuations, even in large arterial and venous vessels [14, 22, 28], pointing to a more complex and intricate nature of physiological effects in the brain. These effects are thus not easily to be separated from neuronal activity when using common, long *TR* measurement protocols.

**Figure 1:**
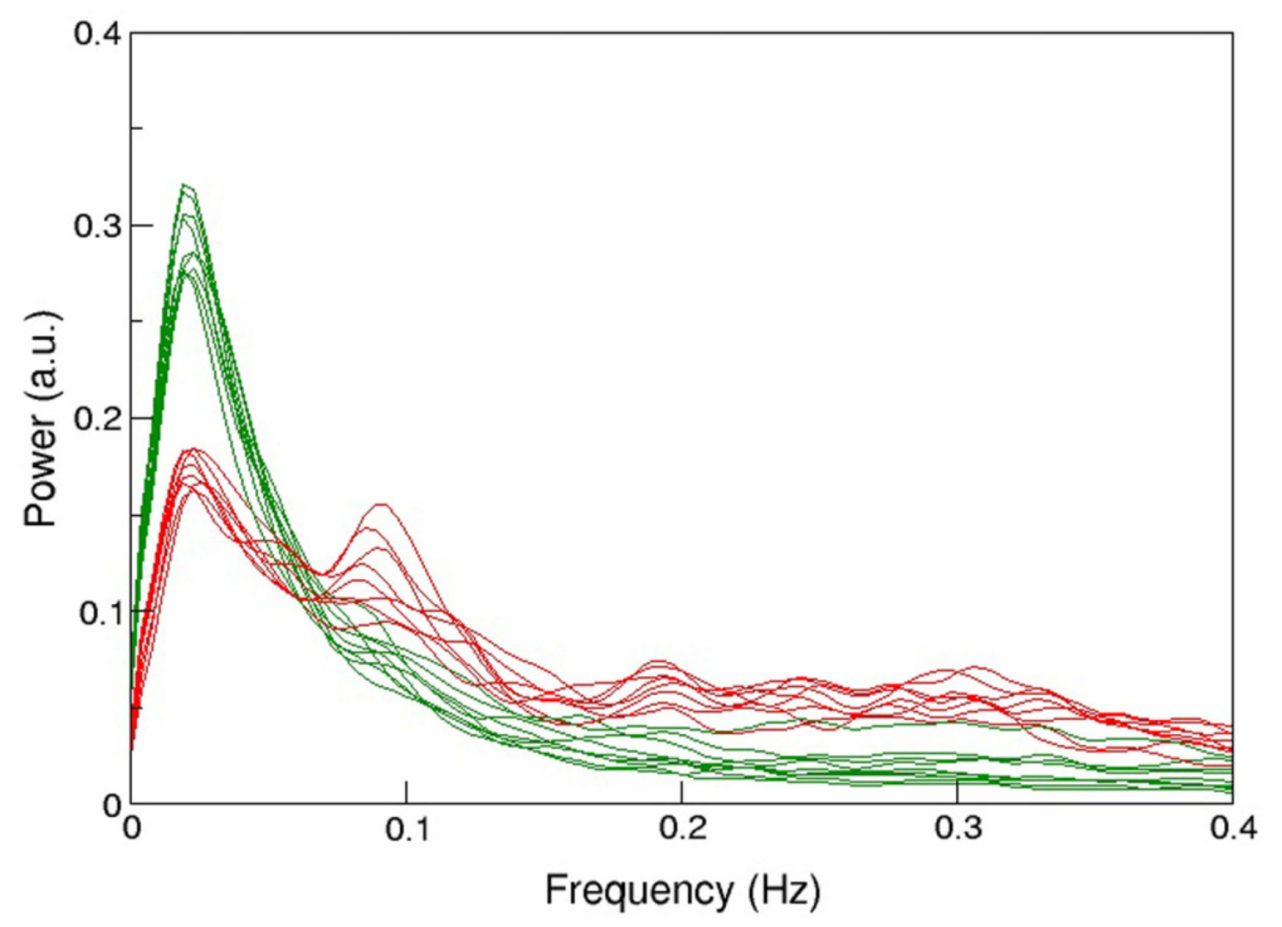
Comparison of the mean spectral distribution of 12 resting-state networks shown in green (i.e., from medial visual, motor, cerebellum, lateral visual, posterior parietal, left-lateral fronto-parietal, temporal, medial frontal, default-mode, to limbic lobe, basal ganglia, right lateral fronto-parietal and anterior temporal lobe, in descending order of explained variance) and various physiological noise components in red. Experiments were performed at 3T (*n* = 26), *TR/TE* = 1000/28 ms (3.3 × 3.9 × 4 mm^3^ voxel resolution) during 5 min, and at 4T 3 (*n* = 15), *TR/TE* =2200/33 ms (3 × 3× 3 mm^3^ voxel resolution) during 10 min sessions. For more details see Robinson [31]. Note the high power of noise components between 0.01 and 0.1 Hz, not to be eliminated via bandpass filtering, and limiting the detection of more resting-state networks or subtle differences between networks in group studies.

**Figure 2:**
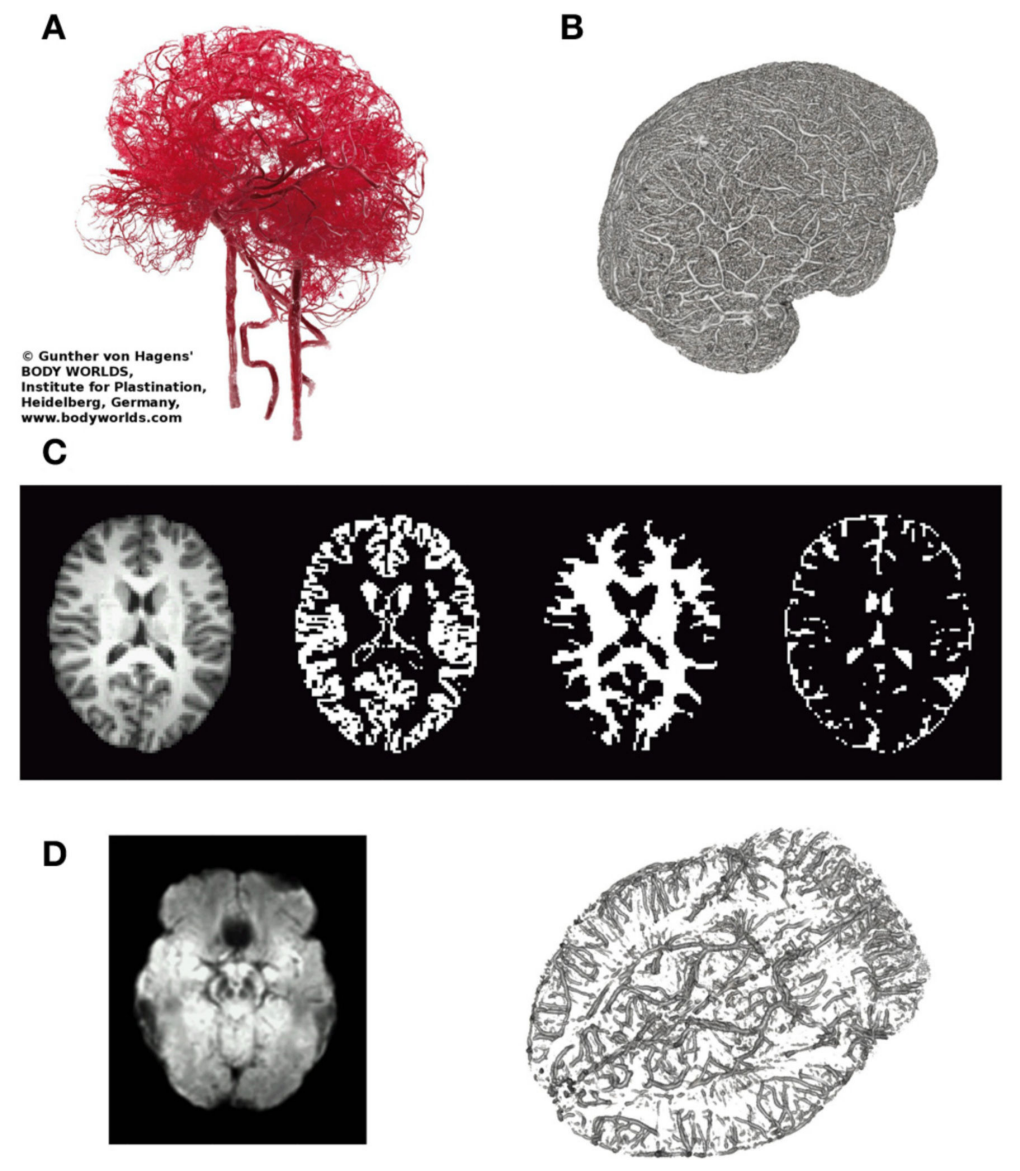
Illustrates relevant macroscopic components in BOLD-based fMRI of the brain. **(A)** Arterial vessels *ex vivo* (copyright Gunther von Hagens, Körperwelten, Institut für Plastination, Heidelberg, www.koerperwelten.de), **(B)** MR-venography of venous vessels *in vivo* at 7 Tesla [courtesy Dr. M. Barth; adapted from Koopmans [36]]. **(C)** Representative slice across the brain of a young healthy subject. T1-weighted structural image, segmented gray matter mask, segmented deep white matter mask and segmented CSF space (from left to right). Note that in contrast to **(A,B)**, vessels are almost invisible. **(D)** Mean SWI (*n* = 3, left), highlighting brain regions with strong susceptibility differences (dark regions) causing artifacts (e.g., frontal lobe/nasal cavities, temporal lobe/ear canals, veins near the brain stem, etc.). MR-venogram (*n* = 1), visualizing the basal vein of Rosenthal, running next to the amygdalae and brainstem.

The protocol recommended to optimize sensitivity and specificity in BOLD-based functional MRI has been summarized recently [29]: the most important parameters in gradient-echo based MR-sequence are echo time *TE* (inversely depending on field strength to match T_2_*), and repetition time *TR* (recommended ≥2 s, motivated by the slow hemodynamic response function, and to allow full brain coverage, also avoiding T_1_-related in-flow effects). However, using this rather long *TR* leads to unknown signal contamination by breathing and heart-beat related physiological noise, ultimately limiting time-series SNR [3, 14, 24, 25, 30]. Robinson et al. [31] have shown that in resting-state fMRI a significant amount (i.e., ≥50%) of high-frequency physiological noise is folded into the very low frequency range (≤0.1 Hz), not to be eliminated via a band-pass filter (see also Figure 1). Also in 2009, they have discussed in detail challenges and potential solutions particularly focused on fMRI of emotions [32], as relevant structures like the amygdalae and the medial-temporal lobe pose severe problems in BOLD-based EPI [33]. In order to visualize important anatomical and physiological aspects of the human brain relevant to our claim, we show representative pictures of the arterial vessel distribution *ex vivo* (Figure 2A), large venous vessel networks *in vivo* (Figure 2B), and segmented brain structures, i.e., gray matter, white matter and CSF, obtained from anatomical scans (Figure 2C). Although vessels are typically not prominent in the anatomical scan nor tissue mask, it is hard to imagine that there would be many brain voxels (size ≥ 2 × 2× 2 mm^3^) not contaminated by large arterial pulsations, draining veins or susceptibility differences, depending on anatomy (Figures 2A,B,D), or any combination thereof [34–36]. This macroscopic complexity level may already help to understand why BOLD-based fMRI is performed best in parieto-occipital brain regions (i.e., small magnetic susceptibility differences and less brain pulsation), as compared to frontal brain regions (increasing magnetic susceptibility differences due to bone-air-tissue borders leading to signal dephasing), and temporo-ventral regions like the amygdala (in addition to strong local magnetic susceptibility differences, these regions are also influenced by brain stem and vessel pulsations in the frequency ranges of 0.13–0.3 Hz and about 1–6 Hz). Intertwined with gross, rigid skull motion (even in the submillimeter range) and respiration artifacts, this may add substantial physiological noise to the measured signal, that cannot be modeled or removed easily when scanning with *TR*’s of 1–5 s [27].

Therefore, the measured signal in any voxel will be composed of several time varying physiological components in the examined region. The predominant signal components mainly rely on the chosen MR technique, corresponding also to alterations of the investigated physiological target (e.g., change of venous oxygen concentration in BOLD imaging). As indicated above, the separation of components truly related to neuronal activity from any other effects remains challenging. While in task related fMRI an informative framework is provided by the paradigm and timing of the experiment for the identification of any specific local brain activation, this framework does not exist for a resting state study. Thus the correlation of temporal signal fluctuations in one cerebral region with another region could potentially depict intrinsic regional physiological effects in the human brain (e.g., capillary pulse wave pulsation, blood flow, chemical tissue properties, etc.), rather than “true” interconnected neuronal activity. Therefore, it is of utmost importance to select MRI methods for a resting state experiment in such a way that enables the differentiation of neuron-specific effects from basic cerebral physiological function in any connective network. This cannot be achieved without a priori knowledge of brain physiology and a multiparametric MRI approach.

A common approach, particularly in neuroscience, is to scan data from a number of more or less well defined subjects and create group averages. Recently it has been claimed and demonstrated that typical group size published might be too low, potentially leading to false conclusions [37], misleading the design and power calculation of subsequent studies [38]. Still, a strong case can be made that a simple increase of numbers does not effectively increase functional contrast-to-noise, at least not as expected. We will demonstrate below that this is mainly caused by the low specificity of many published functional MRI studies, in particular in magnetically heterogeneous regions, also affected by physiological pulsations in the brain, despite attempts to specifically optimize the fMRI protocol [31, 33, 39–44]. In addition, we will show that the major cause of low specificity might be due to strong physiological signals picked up by the measurement technique, particularly in the frequency range <0.1 Hz [45], increasing individual noise levels [27], that cannot efficiently be reduced by the use of high field strength [3, 46], nor by standard correction algorithms [47], or group averaging [48].

Here, we summarize our experience in fMRI, suggest an improved MR-protocol for single-subject data of high quality, with the potential to remove confounding physiological signals from larger vessels, and attempt to stimulate discussion on current limitations and future potential in functional MRI.

## Materials and Methods

### Subjects

Ten healthy subjects (5 females/5 males, mean age = 31.9 year, *SD* = 8.9 year) were recruited at Medical University of Vienna. Exclusion criteria were prior psychiatric or neurologic illnesses, as well as the usual exclusion criteria for MR studies. All subjects gave written informed consent prior to the scan and the study was approved by the local institutional review board.

### Data Acquisition

All MRI scans were performed on a 3 Tesla TIM Trio using the standard 32-channel head coil and whole-body gradients (Siemens Medical Solutions, Erlangen, Germany).

First, a high-resolution anatomical image was acquired using MPRAGE with 1 × 1× 1.1 mm^3^ resolution, and 160 axial slices (*TE/TR* = 4.21/2300 ms, flip angle 9°, inversion time 900 ms). Second, BOLD fluctuations at rest were measured with an advanced, low-*TR* multi-band EPI-sequence [49] using 1.7 × 1.7 × 2 mm^3^ resolution, 2 mm slice gap (matrix size 128 × 128, 32 axial slices, *TE/TR* = 31/333 ms, flip angle 30°, multiband factor 8, bandwith = 1776 Hz/Pixel) collecting 1200 volumes.

For illustration, time-series and image SNR of the multiband sequence (MB4 and MB8, respectively) was compared with a standard EPI sequence (*TR/TE* = 1800/30 ms). For the image SNR, the mean of the voxels within a sphere of radius 5 mm in a given region divided by the standard deviation of the voxels in a sphere of the same size in the air outside of the head was computed for each time point in the dataset, and the mean of these values was given as the image SNR. For the time series SNR, for each of the voxels in the 5 mm sphere, the mean divided by the standard deviation of all time points was computed, and the mean of these values across the voxels in the sphere was given as the time series SNR. For comparison purposes, the SNR values of all regions were normalized by the corresponding SNR from the standard EPI sequence.

### Matching Paradigm

After resting-state measurements, a simple perceptual task previously described by Hariri et al. [50] was performed. Three conditions were used in the paradigm, termed “faces,” “IAPS” and “forms” hereafter. There were four blocks each of the “faces” and “IAPS” conditions, where the images shown to the subjects were standardized emotional faces and unpleasant stimuli from the IAPS picture database, respectively. In each condition, three pictures were shown to the subject. The picture in the top row represents the target, and subjects are required to select which from the two pictures presented in the bottom row is identical to the target picture. In each block, six images are presented sequentially for 5 s each. Between these blocks, the “forms” reference condition was performed where the task remained the same, but emotionally neutral geometric shapes were used instead of pictures. The data was acquired with the low *TR* MB8-EPI sequence as in the resting-state scan, but measuring 1420 repetitions.

### fMRI Preprocessing

All data were preprocessed with a combination of AFNI [51] and FSL [52], using an analysis framework in R [53, 54] on Ubuntu Linux (Version 11.10 “Oneiric Ocelot”). Anatomical images were skull-stripped and normalized to MNI152 standard space. Functional images were corrected for intensity inhomogeneity using a bias field estimation by FSL FAST, skull-stripped and realigned to the 500th volume. Subsequently, functional images were aligned to the anatomical images in MNI152 standard space and resampled to 2 × 2× 2 mm^3^ isotropic resolution, and motion parameters (three translations and three rotations) were regressed out using a generalized linear model (GLM).

### Independent Component Analysis

After preprocessing, voxel time-series were scaled to mean 0 and standard deviation 1. Time concatenated temporal group ICA was performed using *R* were the step of pre-whitening and dimensionality reduction prior to ICA via PCA was computed by an iterative algorithm for singular value decomposition (SVD) developed by Baglama and Reichel [55]. The ICA itself was computed by the fastICA algorithm [56].

### 3D Visualization

The three dimensional visualization of the veins as measured with SWI was done in Slicer [57].

## Results

**Figure 3:**
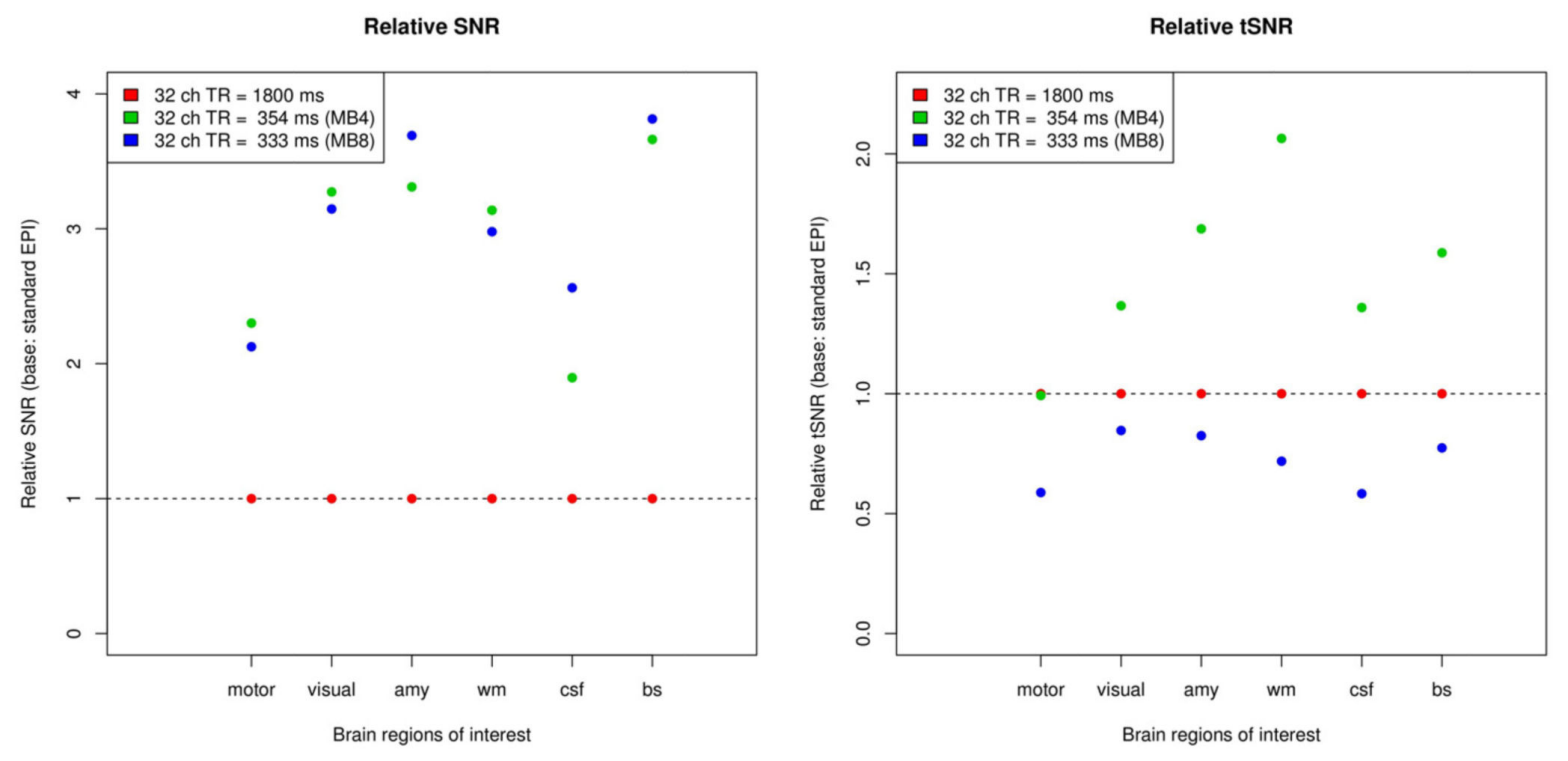
Improving image SNR (left) and time-series SNR (right) via faster scanning. Although SNR per slice or volume is lower at lower *TR*, SNR per unit time is increasing due to more efficient scanning, as compared to *TR* = 1800 ms. Furthermore, time series SNR is also improving, depending however on the brain region or ROI chosen (motor, motor cortex; visual, visual cortex; amy, amygdala; wm, white matter; csf, cerebrospinal fluid; bs, brain stem). Note that while tSNR is increased compared to standard EPI when using a multiband factor of 4, it is however, decreased at a multiband factor of 8.

When comparing time-series and image SNR of multiband EPI with standard EPI measurements (see Figure 3), the most evident difference are the markedly higher SNR values of the former, with image SNR from multiband factor 4 to multiband factor 8 measurements being largely similar. The differences in time-series SNR are less pronounced than the differences in image SNR, but broadly, it can be said that the multiband 4 measurements showed higher time-series SNR than standard EPI, whereas the multiband 8 measurements had slightly lower time-series SNR. Furthermore, it can be seen that SNR increases are more pronounced in the white matter, amygdala and brain stem, and less so in the motor cortex.

**Figure 4:**
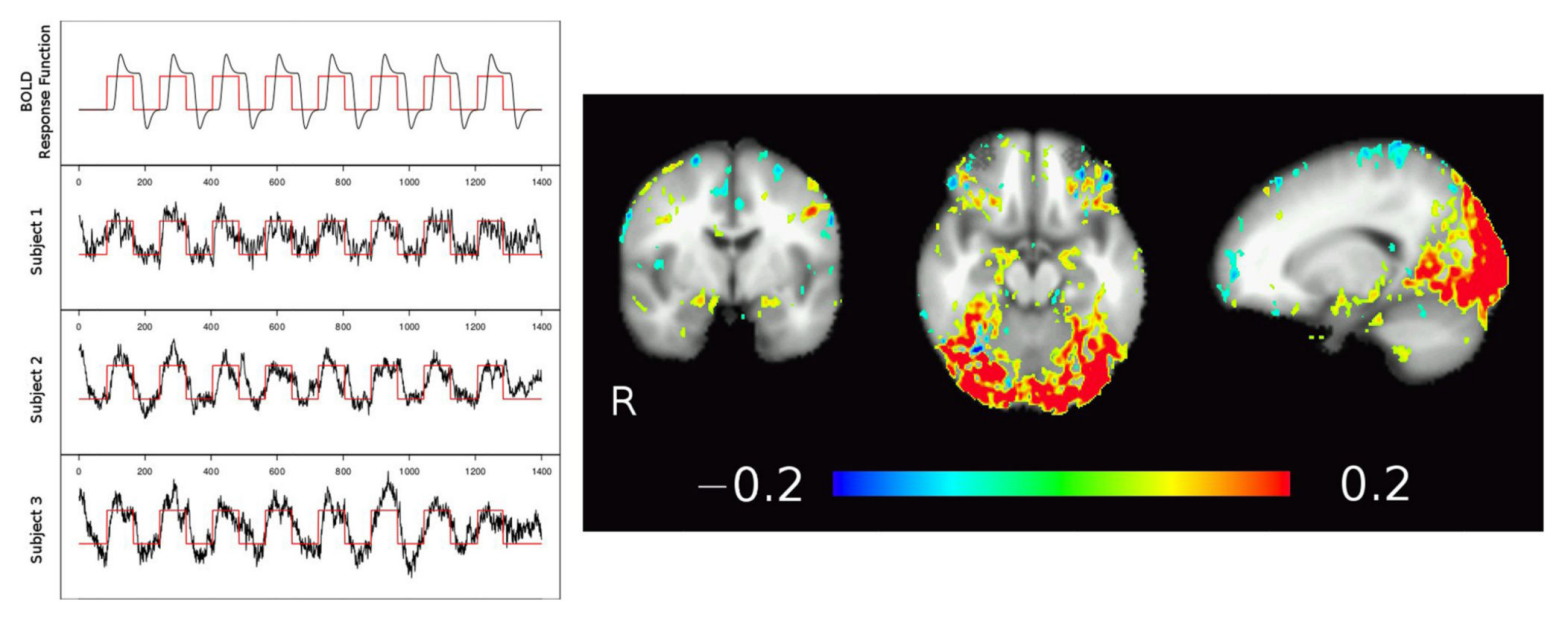
Temporal ICA of low TR, multiband EPI fMRI data from three subjects. Using a strong task (image matching paradigm, block design shown in red), tICA identifies the activation map in the visual cortex but also adjacent to the amygdalae and at the fronto-basis, corresponding to task related time courses as well as strong pulsations (high frequency noise). Note also the major draining vein (V. parieto-occipitalis interna connecting to V. basalis Rosenthal) following medially the temporal lobe next to the amygdalae.

Results from temporal ICA are shown in Figure 4, with time courses of the theoretical BOLD response function (black line in Figure 4, left, top row) modeled by folding the boxcar function of the task blocks (red line; a value of 1 corresponds to a faces or IAPS pictures block, a value of 0 corresponds to the control condition of geometric shapes) with a generic hemodynamic response function. The individual subjects’ BOLD response to the stimuli extracted from the temporal ICA component time-series are pictured in Figure 4 row 2–4 left, and clearly show the inter-individual variability. The corresponding spatial map of that tICA component shows increased signal during the faces and IAPS pictures blocks in the visual cortex and amygdala regions, as shown in Figure 4 right (yellow-red). Note that due to the increased T_1_-weighting (low TR) in the multi-band EPI sequence, also arterial vessels are visible (depicted in blue).

## Discussion and Outlook

The improvements in spatial and/or temporal resolution by multiband EPI sequences developed in the Human Connectome project [44] make it possible to employ tICA in the analysis of fMRI experiments to separate stimulus-related signals from physiological effects of brain pulsations, mainly consisting of high-frequency oscillations.

Two major aspects presented here are the improved fMRI data quality achieved with multi-band as compared to standard EPI sequences, as well as the identification of physiological components, with the potential to selectively remove them from the data.

The first of these aspects also means that the use of low-*TR* multiband EPI entails the possibility of sampling different effects of physiological pulsations on MR signal in the brain. We would like to reemphasize that at a certain point, when *TR* is lower than T_1_ regional cerebral blood flow contributes stronger to EPI-measurements, which was however separated from T_2_* (BOLD) contributions by the presented tICA analysis. Additionally, we found strong evidence that tICA could be useful in separating various highly relevant physiological effects on BOLD imaging related to cerebral blood flow. The perfused human brain and arterial vessels are pulsating in a rather complex fashion, where some effects could appear more prominently when 1/*TR* gets near their respective frequency range. It is well known that at least 4–6 heart beat cycles are required to support a regular perfusion of the brain, obviously depending on cardiac function appearing as heart rate (HR) and heart-rate variability (HRV) in functional brain data (power spectra). In addition, respiration rate (RR) and respiration-rate variability (RRV) may add to physiological “noise” picked up by the fMRI measurement, limiting statistical power in single subjects and group analyses [27, 58]. Furthermore, following current theories [59], the brain may not expand in the same manner everywhere and, consequently, the induced brain motion is also not homogeneous across the whole brain. The major motion component may depend on the brain region and the cardiac cycle phase. In BOLD-imaging variations in the tissue blood- and CSF-content in voxels adjacent to the ventricles are also known to contribute to the signal observed [60]. Thus blood flow, heart beat frequency, consecutive brain pulsation and transmantle stress as well as pulsatile CSF-flow and shift, respectively, should be taken into account in the interpretation of any resting state analysis.

The acquisition of whole-brain datasets with a low *TR* enables researchers to use temporal ICA on these data, a method that could not be used for standard EPI measurements due to convergence issues related to the low number of time points. The combination of low *TR* (i.e.,<500 ms) and ICA leads to a better identification of the task-related HRF, including both its spatial extent and its time course—indeed well enough to examine variations in amplitude, time-to-peak and shape across the brain and between subjects (c.f. 61–63). When applying this method to patient groups, it can thus help to increase specificity and, ultimately, the power to detect significant differences. The increased separability of neuronal and pulsation signals may be due to the greatly improved data quality in low-*TR* MB-EPI, improving average image SNR plus time series SNR per unit time (Figure 3). Furthermore, with increasing image SNR there is great potential to separate physiological noise from neuronal signals via ICA, i.e., to significantly increase functional contrast [64]. This is not achievable with long *TR* data [65] or only statistically across a group of young subjects [64], where physiological noise cannot be separated adequately with spatial—in contrast to temporal—ICA in resting-state data. As Beall and Lowe pointed out, the parallel collection of physiologic data via respiration belt and plethysmograph may no longer be required. However, given the complex physiological mechanisms, which drive the brain, even in resting state, only low *TR* data may fully account for individual variations in heart rate (e.g., arrhythmia) and respiration (e.g., emotion modulated), in particular in patients with or without medication.

On a further note, the identification of pulsation-related high frequency tICA components in itself may also lead to useful applications in the clinical setting, as a disruption of typical oscillation patterns may point to local as well as global vascular and other physiological impairments.

Still, before we achieve adequate spatio-temporal resolution in fMRI, we should be extremely careful not to misinterpret our data [66] and refrain from over optimistic modeling approaches although there are promising ideas [67]. Bringing together basic models developed in physiology and whole-brain BOLD maps [68–70], without taking into account permanently ongoing cerebral blood and CSF circulation [34–36, 71] will prevent full separation of neuronal coupled function from other physiological effects. For instance, the connection seen in Figure 4 between the visual cortex and more anterior regions of the brain, including areas around the brain stem and the amygdala, might not necessarily reflect a pattern of local neuronal activation, but is more likely caused by signal changes in the nearby *V. basiliaris* (Rosenthal) as seen in Figure 2D (transversal SWI slab) and corroborated by many anatomical studies (e.g., 71, Figures 4, 5). Such a connection could result in stimulus-correlated BOLD signal fluctuations in venous vessels running close to the amygdalae, potentially masking any “true” neuronal activity related BOLD signal in the amygdala [72, 73]. As discussed by Turner [17], this may not be a serious limitation in cortical areas—in regions like the amygdala or insula, however, this might be the cause of inconsistent results concerning left and/or right amygdala activation [74–76]. A more detailed study focusing on the amygdalae is currently under way.

Advanced, multiband EPI (available via the Human Connectome Project; 44, 77: nominal voxel size of 2 × 2× 2 mm^3^, and a *TR/TE* = 100–350/30 ms, with multiband-factor 4–16 and GRAPPA 2–3 to cover the whole brain, although high multiband factors and very short *TR* will not allow full brain coverage. We would like to add, however, that this way the Nyquist frequency is increased to >1–5 Hz, i.e., faster signal fluctuations due to respiration, heart-beat, vessel pulsation etc. are now sampled properly and can be identified and eliminated via temporal ICA techniques (45; see Figures 1, 4, 6), further increasing functional contrast, even in resting-state data.

More improvements are expected rather soon, further reducing current technical limitations. However, the most limiting factor is the researcher’s brain itself. Using novel approaches and venturing into the mist of brain function, particularly when attempting to improve our understanding of our own brains, is a quite challenging task. Kahneman [78], in his treatise “Thinking fast and slow,” may provide more hints how to overcome some of the problems. As Kahneman points out, “The difficulties of statistical thinking contribute … to a puzzling limitation of our mind: our excessive confidence in what we believe we know, and our apparent inability to acknowledge the full extent of our ignorance and the uncertainty of the world we live in. We are prone to overestimate how much we understand about the world and to underestimate the role of chance in events”—this fallacy is particularly relevant in a complex and noisy environment like the human brain.
